# Eyewitness identifications after witnessing threatening and non-threatening scenes in 360-degree virtual reality (or 2D) from first and third person perspectives

**DOI:** 10.1371/journal.pone.0238292

**Published:** 2020-09-02

**Authors:** Thomas J. Nyman, Jan Antfolk, James Michael Lampinen, Julia Korkman, Pekka Santtila

**Affiliations:** 1 Faculty of Arts, Psychology and Theology, Åbo Akademi University, Turku, Finland; 2 Faculty of Arts and Sciences, New York University Shanghai, Shanghai, China; 3 Department of Psychological Science, University of Arkansas, Fayetteville, Arkansas, United States of America; University of Padova, ITALY

## Abstract

In eyewitness research the frequent use of video playback presented on a computer screen (i.e., 2D videos) in laboratory-based research is problematic due to the low realism of this method when presenting, for example, threatening (and non-threatening) first-person (and third-person) scenarios. However, in contrast to 2D videos, 360-degree videos presented in virtual reality (VR) presents the opportunity of achieving more realistic and immersive scenarios that might be better suited to mimic real-life incidents, as for example, in the case of a threatening first-person robbery. In the present study, we asked 37 participants to watch eight pre-recorded threatening or non-threatening 2D and VR videos, viewed from either a first- or third-person perspective. After each video, participants assessed the observed target’s appearance and were then presented with either a target present (TP) or target absent (TA) six-person photograph line-up. We expected that VR would result in higher degrees of accuracy in both TP and TA line-ups compared with 2D and that the differences between manipulations would be more pronounced within VR compared with 2D. We found that TP (but not TA) accuracy was higher in 2D compared with VR videos (*91 vs*. *66%*), that there was no main effect of perspective, and that threatening scenes increased TP (but not TA) accuracy compared to non-threatening scenes (*86 vs*. *70%*). Furthermore, in VR (but not in 2D), threatening scenes increased TP (but not TA) accuracy compared with non-threatening scenes (*85 vs*. *40%*). The results go against the expected increased accuracy in VR (vs. 2D) videos but support the notion that threatening (vs. non-threatening) scenes can increase identification accuracy in VR but not necessarily in 2D.

## Introduction

Eyewitness identification evidence of alleged suspects is frequently used by courts and is a highly impactful piece of evidence [1–3]. However, the majority of eyewitness research is limited by the boundaries set by laboratory designs where a common stimulus is a pre-recorded video that is presented on a tv or monitor screen (hereafter referred to as 2D). As for example, in the case of showing a mock robbery, where participants are asked to view, on a computer screen, a pre-recorded event of a perpetrator robbing a cashier. This methodological approach is used due to its low cost and logistical advantages, yet it may have low ecological validity and generalizability to the field [4]. When comparing laboratory-based and live studies it has been suggested that 2D results are comparable with the results from live settings [5,6], yet it has also been suggested that laboratory-based studies underestimate the magnitude of the found effects [4]. Furthermore, when it comes to estimator variables (i.e., contextual factors that cannot be influenced after the event), such as stress [7], distance [8,9], weapon focus [10,11], or attention [12,13], it may not be a question of underestimating the magnitude but, rather, that laboratory results may not correspond with real-life effects of these variables.

For example, stress has been found to have a negative effect on memory and identification accuracy in eyewitness research [7] while at the same time fundamental memory research shows that arousal can positively affect memory consolidation [14]. A relatively recent meta-analysis [15] also illustrates that when a stressor and a stimulus are closely connected this leads to enhanced memory encoding. The somewhat contradictory findings may be due to the video-based method that is frequently used in eyewitness research; a method which might not adequately elicit high arousal closely associated with, for example, a perpetrator in a 2D video [16]. This indicates that in certain cases, such as, when investigating the effects of stress on eyewitness accuracy, the results from a 2D experimental setup might not be consistent with real-life scenarios.

There are, therefore, many important differences between 2D pre-recorded stimuli and live events, which may be significant to the field of eyewitness research. For the purpose of this paper, we will mention a couple of these differences. First, due to the lack of realness in 2D video stimuli, it is unclear if the results (i.e., reactions and outcomes) from presenting a participant with such a stimulus are comparable with those that would be found in a real-life setting. Second, 2D video stimuli rely on a fixed perspective or viewing angle and this does not mimic real life very well, because in real life a person is free to look in many directions. However, an alternative to presenting stimuli in 2D is to present stimuli in Virtual Reality (VR), the use of which has gained momentum in many areas of psychology during the past two decades [17]. This is because VR includes the cost effectiveness, logistic advantage, and high level of control of a 2D video setup, with the added possibility of presenting stimuli in a manner that is potentially more comparable to real life [18]. Moreover, although VR has earlier been a high-cost method, it is now a reasonably low-cost technology that has the capacity to produce high quality experiences [19].

VR is used in order to re-create so called “realness effects” and “the sense of being there” [20]. VR and 2D are similar in the amount of experimental control they allow, but the advantage of VR is that it is an immersive technology built to draw the user into the virtual world [21]. This is achieved by either filming a scenario with a 360-degeree camera and presenting it in VR or creating a 3D scenario using, for example, the game engine Unity and presenting it in VR (for a forensically relevant VR study, see [22]). In order to present a virtual world to a participant, it is possible to use alternative methods, such as, a VR head mounted display (HMD) or a projection-based display (a so called “cave” setup). In the present study we used an HMD and for the sake of brevity we will focus only on describing how VR is presented with an HMD.

When presenting a 360-degree pre-recorded live scenario or a 3D computer-generated scenario, an HMD is placed on the participant’s head, and by using individual lenses for each eye, the virtual world is presented to the participant. This means that in VR the participant’s total vision (i.e., both central and peripheral vision) is occupied by the stimulus material (in contrast to a traditional 2D monitor or tv screen where only the central vision is occupied by the screen and the room where the participant is sitting occupies the participant’s peripheral vision). Furthermore, the HMD allows the participant to shift perspective in the scene by moving their head [18]. In other words, VR potentially mimics real life more accurately compared with 2D because in VR both central and peripheral vision are occupied by the recorded/created stimulus and it is possible to look in any direction. This implies that an investigation of, for example, how different estimator variables affect attention, could potentially be more generalizable to real life when using VR compared to 2D.

Realness and believability are also crucial aspects related to the ecological validity of eyewitness research and it is difficult to create a believable 2D scenario depicting, for example, a threatening first-person perspective. However, first-person threatening scenes are common criminal events in real life and it is in such a situation that there is reason to believe that memory encoding could be enhanced rather than weakened by a threatening stimulus [23]. Here, VR has the potential to immerse the participant in a scene where a first- and third-person perspective can be manipulated in a realistic manner and where the first-person perspective can “feel” real enough to be experienced as a threatening situation.

## The present study

We investigated eyewitness identification and rejection accuracy after viewing video stimuli presented on a computer screen (i.e., a video recorded with a standard camera and presented on a computer screen; 2D video) or in VR (i.e., a video recorded with a 360-degree camera and presented through an HMD in VR). We created both a threatening (i.e., a robbery with a weapon) and non-threatening (i.e., no robbery and no weapon) video stimuli. Moreover, we also filmed the threatening and non-threatening scenes from a first-person (i.e., victim) and a third-person (i.e., onlooker/witness) perspective (for an illustration of the different conditions, see S1 Appendix). Moreover, this study was designed as a proof of concept study and for this reason, we recruited only a small sample and did not include cumbersome measures of physiological responses (see also limitations).

Based on the theoretical background, we expected there to be a main effect of the VR and 2D manipulation, manifested by VR giving rise to a higher degree of target present (TP) identification (i.e., correctly identifying the perpetrator/target when they are present in the line-up) and target absent (TA) rejection accuracy (i.e., correctly rejecting the line-up when the perpetrator/target is not present in the line-up) compared to 2D. The underlying assumption here was that because VR scenarios would be perceived as more real (compared to 2D), they would be inherently more attention-capturing and would result in a higher degree of focus on the faces and higher degree of memory encoding (compared to 2D). Secondly, we expected to find a main effect of threat on TP identification and TA rejection accuracy, with threatening scenes eliciting more correct decisions compared to non-threatening scenes. This prediction was based on the fact that as the threat was operationalized as the perpetrator acting threateningly (i.e., the threat was directly associated with the stimulus), we expected that memory encoding would be enhanced by threatening scenes in accordance with the meta-analysis by Shields and colleagues (2017). Thirdly, we expected there to be a main effect of perspective, with a first-person perspective resulting in higher TP identification and TA rejection accuracy compared to a third-person perspective. This third hypothesis was based on the notion that when the scene is directly related to oneself (i.e., a first-person perspective) then increased engagement and attention will have a positive effect on memory encoding. Lastly, we expected that the differences between threatening and non-threatening scenes as well as the differences between first- and third-person scenes would be more pronounced in VR compared to 2D. This was due to the underlying assumption that VR would produce a higher degree of realness and believability compared with 2D.

## Method

### Participants

The sample included 37 university student participants who all made eight eyewitness identifications each. All were recruited through email or via social media. From the 296 identifications, we excluded 28 observations because the participant recognized one or more of the images used in the line-ups. In other words, we had included a post line-up question where we asked participants if they recognized any of the faces prior to the experiment and we used this question to exclude line-up responses where participants had recognized one of the line-up images used (i.e., a familiarity control question).

Thus, the final sample included 268 observations from 37 participants. Nineteen of the participants reported their nationality as Finnish and 18 reported that they were from other countries. There were 21 male participants (*mean age* = 25.6, *SD* = 4.7) and 16 female participants (*mean age* = 23.5, *SD* = 8.6).

### Ethics statement

All aspects of the proposed research project were conducted in accordance with the Declaration of Helsinki. That is, participants were informed of the voluntary nature of the studies, and of their right to terminate their participation in the research at any stage, without giving a reason. Written and full informed consent was obtained from all participants. Research permits were sought and approved from IRB of the Department of Psychology at Åbo Akademi University.

### Stimuli

We filmed eight separate targets (i.e., perpetrators) that acted in all eight conditions of our factorial design. The total duration of each of the scenes was approximately 10–15 seconds. The threatening condition included a target that stole a money-collection jar while threatening the salesclerk with a knife over a table/counter. In the non-threatening condition, the target placed money in the money-collection jar while holding a booklet in the same hand as the knife was in the threatening scene. In the first-person perspective, the point of view was that of the victim, and in the third person perspective, the point of view was that of an onlooker of the two people by the counter (i.e., the victim and the perpetrator). For still images from the video scenes used in the threatening (non-threatening) and first-person (third-person) scenarios, see S1 Appendix. The conditions were filmed using a Canon camera (for the 2D videos) and a Ricoh Theta Z 360-degree camera (for the 360-degree VR videos). During the experiment, the 2D videos were presented on a desktop computer monitor (Dell U3014, screen: 75.6 centimeters; cm (30 inches; in), resolution: 2560 x 1600 pixels; px) and the 360-degree videos were displayed in virtual reality using an HMD. The HMD was an Open Source Virtual Reality (OSVR) HDK 1 (Screen size: 1920x1080 px; height 12.2 cm (4.8 in.), width: 6.8 cm (2.7 in., per eye 1080 x 960 px, width 6.8 cm (2.7 in.), height 61mm (2.4 in.), with a field of view of 100 degrees and a refresh rate of 60 Hz).

The position of the 360-degree camera when recording the first-person perspective was 1 meter from the perpetrator and the camera lens was at a height of 1.5 meters from the floor. Since the 360-degree lens is a fisheye it warps distance to a certain extent (see for example the still images in S1 Appendix) so we had to move the Canon camera to a position of 4 meters from the perpetrator (same height) in order to create 2D recordings where the perspective was as similar as possible to the 360-degree recordings. This was done through trial and error prior to recording all the video scenes, we tested the perspective that most closely matches one another when viewing the scene on a computer screen and when viewing the scene in VR. When we filmed the third-person perspective, we placed the 360-degree camera at a diagonal angle 1.2 meters relative to the target/perpetrator, and we placed the Canon camera at 4.8 meters to the side of the target/perpetrator. The static images of the 360-degree video (see S1 Appendix) do not do justice to the point of view when using the HMD and experiencing the scene in VR; with the HMD on, the 360-degree perspective is much more similar (i.e., the perspective is closer) to the 2D perspective than what it appears from the still images. Furthermore, although the video quality is slightly lower in the 360-video compared with the 2D video, the distance to the face is small and, in all videos, it was easy to discern details of the face of the target/perpetrator, so we considered the difference to be negligible in relation to the line-up task.

The targets that were included in the video scenes were native Finnish university students. There were four female targets and four male targets, whose average age was 23.5 years (*SD* = 4.7), average was height 173.9 cm (*SD* = 8.2), and average was weight 68.3 kg (*SD* = 9.5).

### Materials and measures

We used a Lenovo Tab 2 A10-70 10.1" Android tablet in order to present line-ups and collect participant responses. At the start of each session, participants were asked to fill in demographic information (i.e., nationality, age, and own height) and prior to each line-up they were asked to answer five pre-programmed questions regarding the target’s appearance (not relevant to or analyzed in the present paper), followed by a line-up where identification or rejection of the line-up was achieved by selecting an image or selecting the option to reject the line-up. The tablet also recorded post-line-up questions regarding the participant’s certainty regarding their line-up choice and questions regarding their familiarity with any of the images used in the line-ups (for the sake of exclusion).

The line-up task was a computerized six-person photograph line-up that was randomized (per trial) to be either target present or target absent. The target present line-up included one target image and five filler images, and the target absent line-up included one foil image (i.e., target substitute) and the same five filler images as in the target present line-up. Identifications and rejections were coded as binary outcomes (0/1) by the software application. Identification accuracy was coded as “1” for target present (TP) target identifications, “0” for TP foil identifications, and “0” for TP rejections. Rejection accuracy was coded as “1” for target absent (TA) rejections and “0” for foil/innocent identifications. Confidence was registered on a continuous scale between 0–100% for confidence and response time in milliseconds. Descriptive estimates of the targets were registered as follows: Height (0–220 cm), Target Weight (0–150 kg), Target Age (5–99), Target Gender (Man or Woman), and Distance to target (0–200 meters).

### Procedure

The experimental procedure entailed 1) reading and signing an informed consent, 2) watching eight pre-recorded videos (each video was approximately 10–15 seconds), 3) answering questions and conducting a line-up tasks on an Android tablet. We pseudo-randomized the order of the conditions in our design, so that there were eight possible sequences of our eight conditions. Each participant was allocated to one of the eight sequences, which included eight separate videos and each of the videos included one of the eight targets. This meant that every participant saw eight scenes and conducted eight line-up tasks where one of the eight targets was presented (no one saw the same target twice). The participants either first saw four VR scenes and then four 2D scenes or vice versa (depending on the sequence that they had been placed into). In VR, the participants saw two threatening and two non-threatening scenes, where two were from a first-person perspective and two were from a third-person perspective (the same applied to the 2D scenes). The threatening and non-threatening and the first- and third-person perspective scenes were counterbalanced within the four VR and 2D scenes. After watching each video scene, participants were asked to estimate, by answering questions on a tablet, the target’s age, gender, height, and weight, as well as the distance to the target. This was then followed by a line-up identification task. Lastly participants were asked to make a confidence judgment (i.e., the self-perceived certainty with which decisions were made) and to state if any of the images used in the line-up were familiar from prior to the experiment.

### Design

The present experiment was a within-subject factorial design with three factors: two (2D or VR) by two (threatening or non-threatening) by two (first- or third-person).

### Statistical analyses

The main analyses were conducted using the *R* platform [24] and by fitting data as a multi-level logistic regression in the *lme4* package [25]. The general formula used was:

Outcome∼Predictor(s)+(1|subject)+(1|Target)

In *lme4* the categorical variables are by default contrast coded and the outcome of the binary logistic regression analysis is a value of log odds [26]. This value is used to assess the likelihood of a response being 1, using the *probit* function (exp(x)/(1+exp(x)), where x is the log odds estimate of interest. The multilevel model (also known as random effects model), is an appropriate approach of analyzing the current data as it takes into account the hierarchical nature of our repeated measures design and includes the within and between subject variance as part of the statistical model. Due to the limited number of observations per cell (in the experimental design), we were not able to run full factorial analyses, using all three predictors and their interactions in the same model. For this reason, we analyzed the results sequentially, by first looking at main effects and then their interactions (including planned comparisons). This resulted in six separate analyses for TP identification accuracy and six separate analyses for TA rejection accuracy.

Based on the log odds from the logistic regression we also report the odds ratios (OR) and their confidence intervals for the analysis of main effects. Odds ratios are a common way of reporting effect sizes [27]. We also ran six separate post hoc power analyses for each of the analysis of main effects (i.e., three for the TP and three for the TA analyses). For each of these analyses, we used OR = 1.68 as an effect size of interest (comparable to Cohen’s d = .2) [27]. We did this in order to estimate the power to detect a small effect in either TP or TA line-ups when investigating main effects. Here, we used the *powerSIM* function that is part of the *simr* package in r [28] in order to calculate the power (1-β) and confidence intervals (CI), using 200 iterations, to detect a small effect (i.e., OR = 1.68). As can be seen in the results section, the power for each main effects analysis was low, which increases the risk of a type II error in each case, but as this was a proof of concept design, we argue that the results are still of interest for future studies.

In order to conduct an exploratory analysis addressing also the issue of confidence, we created two new confidence-accuracy variables for TP and TA line-ups. Here, we recoded the binary outcome variable 0/1 as -1/1 and multiplied by the post line-up confidence rating and divided by 10. This gave us a continuous variable where 0 to -10 represented the low to high confidence ratings for incorrect decisions in TP line-ups (i.e., filler selections or rejections) and TA lineups (i.e., filler selections). Correspondingly, the values 0 to 10 represented low to high confidence ratings for correct decisions in TP line-ups (i.e., target selections) and TA lineups (i.e., line-up rejections). This provided us with a more sensitive measure combining confidence with accuracy.

The data and R markdown script used to analyze that data can be found at the Open Science Framework at the following address: https://osf.io/gz3wy/

## Results

### Descriptive results

Out of the total 268 eyewitness responses, there were 138 TP decisions (VR: 71, 2D: 67), 130 TA decisions (VR: 61, 2D: 69); see Table 1. See also Table 2 for the average post line-up self-reported confidence and the average line-up response times.

**Table 1:** The distribution of correct and incorrect responses by experimental condition.

Condition	TP identifications								TA Rejections					
	n	Target	Filler	Reject	n	Target	Filler	Reject	n	Reject	Filler	n	Reject	Filler
	Threat				No threat				Threat			No threat		
2D First-person	20	18	1	1	17	17	0	0	17	13	4	14	10	4
VR First-person	25	21	2	2	14	6	1	7	8	6	2	18	14	4
2D Third-person	10	9	0	1	20	15	2	3	23	15	8	15	8	7
VR Third-person	16	11	2	3	16	7	1	8	19	15	4	16	11	5

Threat = Threatening pre-recorded scenarios, No threat = Non-threatening pre-recorded scenarios. 2D = pre-recorded video created with a Canon camera. VR = pre-recorded video created with a Ricoh Theta Z 360-degree camera, Target = Target selections, Filler = Filler selections, Reject = Line-up rejections.

**Table 2:** The average confidence and response times by experimental condition.

Condition	Confidence (0–100%)				Response time (seconds)			
	Mean	SD	Mean	SD	Mean	SD	Mean	SD
	Threat		No threat		Threat		No threat	
2D First-person	77.5	24.3	71.4	26.7	10.8	8.5	10.9	13.9
VR First-person	73.4	26.4	68.0	27.2	12.6	11.2	13.1	10.0
2D Third-person	69.3	23.6	66.5	26.3	15.0	19.6	11.6	7.8
VR Third-person	63.3	26.8	66.8	24.8	13.0	8.4	14.4	8.3

Threat = Threatening pre-recorded scenarios, No threat = Non-threatening pre-recorded scenarios. 2D = pre-recorded video created with a Canon camera. VR = pre-recorded video created with a Ricoh Theta Z 360-degree camera.

### Target present identification accuracy

**Fig 1:**
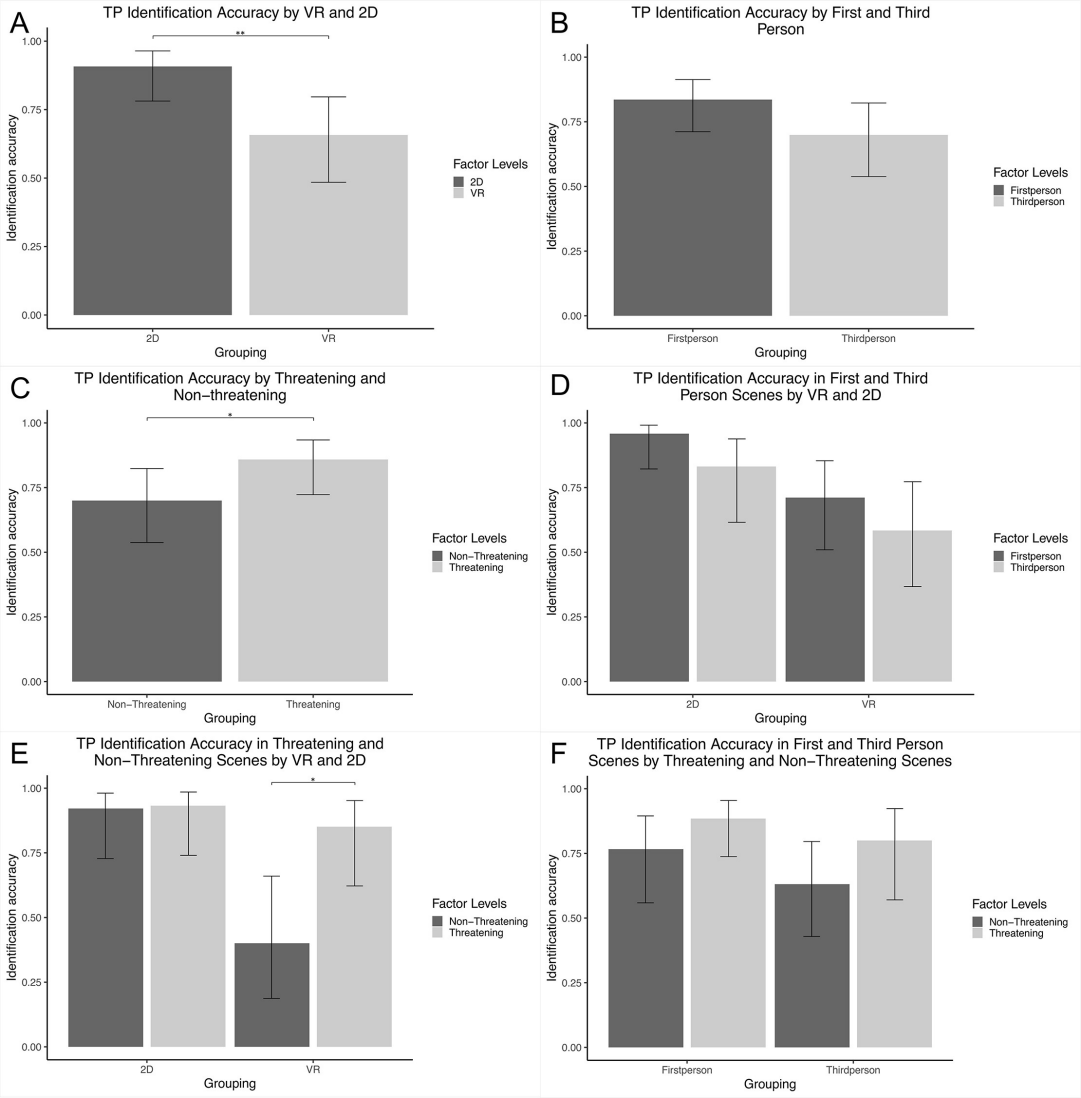
Target present identification accuracy. The error bars represent the upper and lower 95% confidence levels. All means displayed represent the estimated means from multilevel logistic regressions. (A) TP identification accuracy by VR and 2D. (B) TP identification accuracy by First and Third person scenes. (C) TP identification accuracy by Threatening and Non-threatening scenes. (D) TP identification accuracy in First and Third Person Scenes by VR and 2D. (E) TP identification accuracy in Threatening and Non-Threatening Scenes by VR and 2D. (F) TP identification accuracy in Threatening and Non-Threatening Scenes by First and Third Person Scenes. Planned comparisons revealed (E) that there was a significant difference between threatening and non-threatening scenes in VR (*B* = 2.14, *SE* = 0.79, p = .003). No other planned comparisons were significant. Exploratory comparisons revealed that there was (D) a significant difference between first person VR and first person 2D (*B* = -2.24, *SE* = 0.86, *p* = .047) and between first person 2D and third person VR (*B* = 2.80, *SE* = 0.88, p = .008). There was also (E) a significant difference between threatening 2D and non-threatening VR (B = 3.03, SE = 0.89, p = .004) and between non-threatening VR and non-threatening 2D (B = 2.86, SE = 0.90, p = .008). ** = 0.01; * = 0.05.

We found an effect of our VR and 2D manipulation on TP identification accuracy (see Fig 1A), where 2D resulted in more correct identifications compared to VR (*B* = 1.63, *SE* = 0.51, *p* = .001, OR = 5.12 (95% CI: 1.88–13.91), 1-β = 26.00% (95% CI: 20.07–32.66). We found no effect (see Fig 1B) of our perspective manipulation on TP identification accuracy, where a third-person perspective did not differ significantly from a first-person perspective (*B* = -0.78, *SE* = 0.43, *p* = .072, OR = 0.46 (95% CI: 0.19–1.07), 1-β = 19.00% (95% CI: 13.81–25.13). We also found an effect of threatening vs. non-threatening scenes on TP identification accuracy (see Fig 1C), with non-threatening scenes resulting in less correct identifications compared to threatening scenes (*B* = -0.96, *SE* = 0.45, *p* = .032, OR = 0.38 (95% CI: 0.16–0.92), 1-β = 16.50% (95% CI: 11.64–22.38). The results do not support our hypothesis that VR (compared to 2D) results in a higher degree of identification accuracy; rather the results suggest that the reverse is true. We also did not find support for our hypothesis that a first-person (vs. third-person) perspective gives rise to a higher degree of identification accuracy. However, we found support for our hypothesis that threatening (vs. non-threatening) scenes leads to higher identification accuracy.

Investigating interactive effects, we found no significant interaction (see Fig 1D) between the 2D vs. VR and first- vs. third-person perspective on TP identification accuracy (χ^2^ [5] = 0.91, *p* = .339). Planned comparisons revealed no significant differences between the perspectives within 2D or VR (see Fig 1D for exploratory difference between conditions). The results go against our hypotheses that the difference between the first- and third-person perspectives would be more pronounced in VR compared with 2D. We found no significant interaction (see Fig 1E) between 2D and VR and threatening and non-threatening on TP identification accuracy (χ^2^ [5] = 2.56, *p* = .110). Planned comparison, however, revealed a significant difference between the threatening and non-threatening conditions in VR, where the threatening conditions resulted in higher accuracy compared with the non-threatening condition (see also Fig 1E for the exploratory differences between conditions). This falls in line with our hypotheses that threats in VR are more realistic and that threatening scenes enhance accuracy. However, as can be seen in Fig 1E, there appears to have been a ceiling effect of accuracy in both the threatening and non-threatening condition in 2D. It is, therefore, unclear if the lack of difference in 2D was due to the ceiling effect or the actual condition. When we investigated the interaction between first- and third-person perspectives and threatening and non-threatening manipulations on TP identification accuracy. We found no significant interaction between the manipulations (χ^2^ [5] = 0.00 *p* = 1.000) and planned comparisons revealed no significant differences (see Fig 1F). The results indicate that there is no difference between threatening and non-threatening scenes seen from a first- or third person perspective, which goes against our hypotheses. However, this may also be due to overall ceiling effects in the study; indicating that the task was perhaps too easy. Lastly, using the variable combining accuracy and confidence information, we found that the results exactly mirrored the analyses using the dichotomous outcome variable in terms of significant effects.

### Target absent rejection accuracy

**Fig 2:**
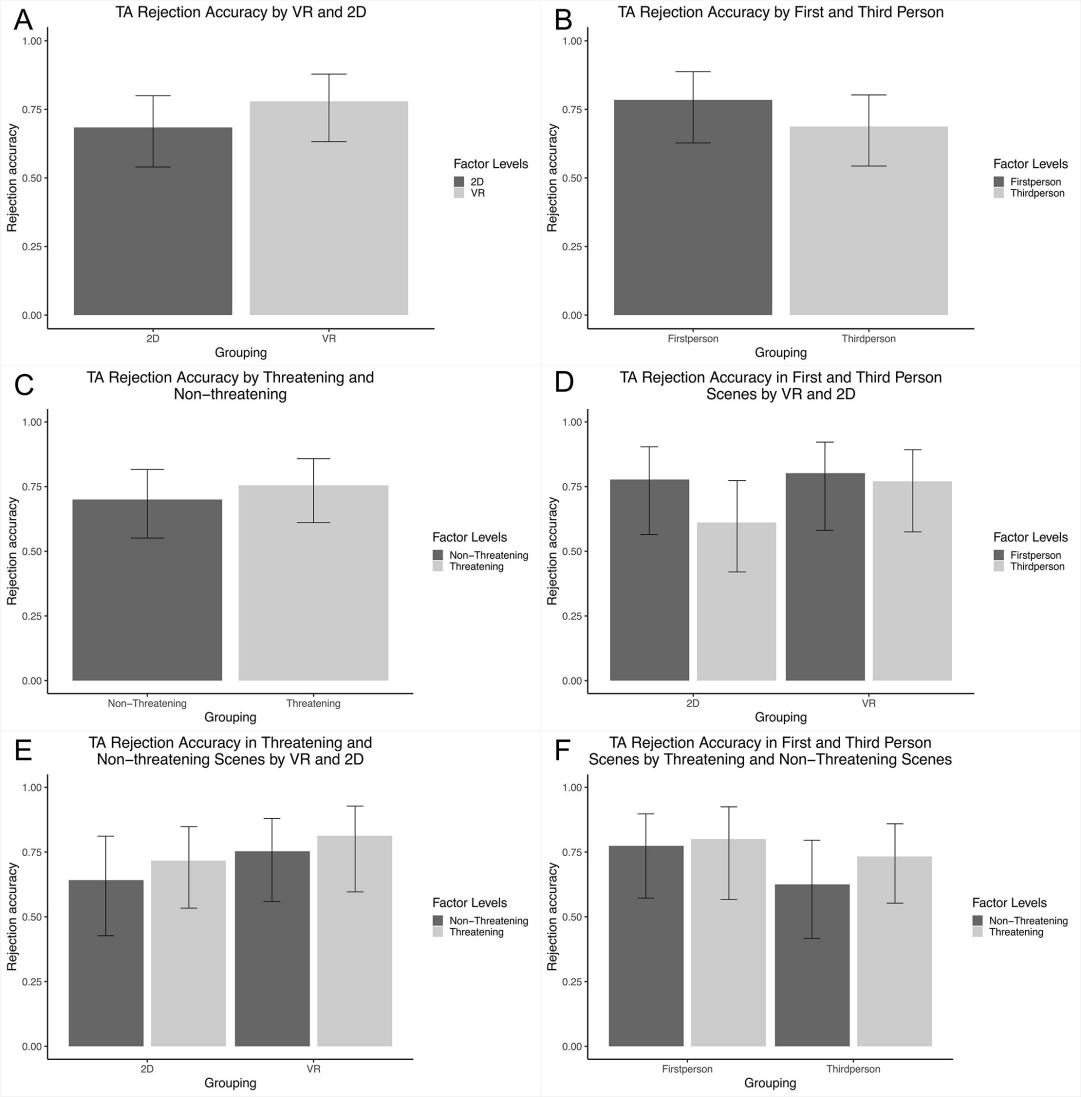
Target absent rejection accuracy. The error bars represent the upper and lower 95% confidence levels. All means displayed represent the estimated means from multilevel logistic regressions. (A) TA rejection accuracy by VR and 2D. (B) TA rejection accuracy by First and Third person scenes. (C) TA rejection accuracy by Threatening and Non-threatening scenes. (D) TA rejection accuracy in First and Third Person Scenes by VR and 2D. (E) TA rejection accuracy in Threatening and Non-Threatening Scenes by VR and 2D. (F) TA rejection accuracy in Threatening and Non-Threatening Scenes by First and Third Person Scenes.

We found no effect (see Fig 2A) of our VR and 2D manipulation on TA rejection accuracy, where 2D did not significantly differ from VR (*B* = -0.49, *SE* = 0.42, *p* = .247, OR = 0.61 (95% CI: 0.27–1.40), 1-β = 18.00% (95% CI:12.94–24.04). Nor was there an effect (see Fig 2B) of our first- and third-person perspective manipulation on TA rejection accuracy, so that the third-person perspective did not differ significantly from the first-person perspective (*B* = -0.50, *SE* = 0.43, *p* = .248, OR = 0.60 (95% CI: 0.26–1.42), 1-β = 23.00% (95% CI: 17.36–29.46). We found no effect (see Fig 2C) of our threatening and non-threatening manipulation on TA rejection accuracy, where non-threatening scenes did not differ significantly from threatening scenes (*B* = -0.28, *SE* = 0.42, *p* = .506, OR = 0.76 (95% CI: 0.34–1.71), 1-β = 19.50% (95% CI: 14.25–25.68). None of the results support our hypotheses.

Investigating the interactive effects, we found no significant interaction between 2D and VR and first- and third-person perspective on rejection accuracy (χ^2^ [5] = 0.49 *p* = 0.486) and no significant interaction between the 2D and VR and threatening and non-threatening manipulations on rejection accuracy (χ^2^ [5] = 0.00 *p* = 0.992). There was also no significant interaction between first- and third-person perspectives and threatening and non-threatening manipulations on rejection accuracy (χ^2^ [5] = 0.15 *p* = 0.696). As can be seen in (Fig 2D–2F), planned comparisons did not reveal any significant differences within groups. The results illustrate that we were unable to find support for any of our hypotheses in relation to TA rejection accuracy and this is in contrast to the findings of TP identification accuracy. Lastly, using the variable combining accuracy and confidence information, we again found that the results exactly mirrored the analyses using the dichotomous outcomes in that there were no significant effects.

## Discussion

The present study was designed in order to investigate the differences between 2D and VR by presenting pre-recorded scenes from either a first- or third-person perspective and the content of which was either threatening or non-threatening. The design was created in order to examine differences between a method that is potentially closer to a real-life setting (VR) with a method that is potentially more removed from a real-life setting (2D). This was based on research showing that VR can be used to create high degree of realness and believable events [18]. Moreover, due to questions concerning underestimated effects from laboratory-based 2D studies [4] and the suggestion that a 2D method might not be a suitable for investigating effects of stress on eyewitness accuracy [16], we were interested in creating a proof of concept design that would be more comparable with a real life setting.

Our design entailed a setup where each participant saw eight separate pre-recorded videos (four in VR and four in 2D), which were either threatening or non-threatening and recorded from either a first- or a third-person perspective. Furthermore, each participant was presented with a computerized six-person line-up that was programmed to randomly present either a simultaneous target present (TP) or target absent (TA) line-up where images were presented in random order. Each participant was asked to first estimate the age, gender, height, weight, and perceived distance to the target in the video, and (when viewing the line-up) to either select and image or reject the line-up. Our hypotheses were that VR would result in higher degrees of accuracy in both TP and TA line-ups compared with 2D and that the differences between manipulations would be more pronounced within VR compared with 2D.

We found an effect of 2D and VR manipulation and an effect of threatening and non-threatening scenes on TP identification accuracy (but not TA rejection accuracy). We found no effect of our first- and third-person perspective manipulation on TP identification accuracy or TA rejection accuracy. Furthermore, our planned comparisons only gave rise to one significant comparison and that was between first- and third-person scenes within VR on TP identification accuracy; where a first-person perspective gave rise to a significantly higher degree of accuracy compared with non-threatening scenes.

The main results of this study go against our hypothesis that VR would results in overall higher accuracy compared with 2D and in only one case, comparing accuracy in threatening and non-threatening scenes within VR, did we find support of our hypothesis that our manipulation would be more pronounced within VR.

There are a number of interpretations that can be made regarding these finding. First, in order create a VR scenario where a person feels and acts as in real life, it is important to make sure that both place illusion and plausibility are effective. Place illusion is a more perceptual process and creates the feeling of being in a scenario even though you know you are not, whereas plausibility (a more cognitive process) is the feeling that what is happening to me is actually happening even though I know it is not [21]. Both place illusion and plausibility need to succeed in order to generate reactions from participants that mimic real life scenarios. Because our pre-recordings were short (10–15 seconds) it is probable that we were unable to achieve either place illusion or plausibility, which means that the lack of immersion may even have had a negative impact on attention, because it was not believable and took away attention from the task. In comparison, a 2D method does not have similar requirements and may therefore have been an easier task. However, our goal was to compare similar scenarios in 2D and VR, so our aim was not to create the most realistic scenario possible in VR, but rather to investigate the potential of using a VR method. Future research should focus on creating longer and more immersive scenarios that mimic a real life setting to a high degree (and compare this with a live event).

Second, assuming that the possible lack of place illusion and plausibility did not directly affect the results, then it is also possible that the lower degree of overall accuracy actually reflects an important difference regarding attention between VR and 2D in this context. That is, that in VR it is possible to be distracted by or tempted to look at irrelevant aspects of a scene because one has more freedom to look around in all directions, whereas is 2D more focus and attention (due to one’s central vision being fixed on the screen and one perspective) is placed on the stimuli. Naturally, all participants were asked to focus on the task at hand in both settings, but there still exists a fundamental difference between a fixed 2D screen and the immersive experience of a VR environment. The lower accuracy might, therefore, represent a crucial aspect that is relevant to real life setting and that is missing in 2D settings. However, it may also be that the novelty of using VR (in contrast to viewing the scenes through a computer screen) may have also distracted the participants to a certain extent. Third, accuracy was on average very high, which might reflect that the task was too easy and that is why we were unable to find larger differences between conditions. Nevertheless, looking at the accuracy levels and the main effects, many follow the overall trend suggested by our hypotheses, which lends support to the notion that more research is needed to further investigate these manipulations in VR and 2D (and a live setting).

### Limitations

Due to the complexity of our design and the small sample size there were a relatively low number of observations per cell (in the factorial design), which did not allow for an analysis of the complete design, which might have revealed interesting interactive effects. Clearly there is need for further investigation with and increased sample size because low statistical power means that it is difficult to interpret both the effects found and the lack of effects. It also appears that the task was too easy, which resulted in a ceiling effects and this also limits the interpretation of the results. However, accuracy rates vary greatly between studies depending on the stimulus material used, such as, live, video, photo material, and the line-up type used, such as, simultaneous or sequential line-ups [29]. In other words, although accuracy was high it was not outside the range of what has been seen in earlier experimental eyewitness studies. A more difficult task would most likely be more adequate when investigating a similar design. Moreover, we did not include objective measures of stress and that limits the interpretation of the current design, which future studies should aim to include.

### Practical implications and future research

The practical implication of this research is that it was an attempt to investigate the potential of using 360-degree videos and VR as a more ecologically valid method compared to using standard 2D monitors. The generalizability of our results is limited by the parameters set by this small proof of concept study; however, the results indicate that VR might be better suited to investigate first- vs. third-person perspectives. There are also many more questions that can be combined with the first- and third-person perspective that are crucial to eyewitness research. For example, in eyewitness research it is generally accepted that stress negatively impacts eyewitness memory and identification accuracy [7], yet this is contrary to basic memory research that states that encoding is improved by stressful compared to neutral events [14]. The connection between stress and memory has been further elaborated on in a recent meta-analysis by Shields and colleagues (2017), where it was found that stress during encoding improves memory when a stimulus is closely associated with a stressor. Moreover, a recent eyewitness study by Sauerland and colleagues (2016) concluded that one of the possible reasons for the differences in findings regarding memory and stress between research paradigms is that eyewitness research has relied so heavily on laboratory-based 2D video stimuli that give rise to only small elevations in stress. The implications are that 2D video is lacking in both realness and immersion and this means that 2D video fails to elicit emotional reactions that are comparable to real life, which in turn leads to small effects. Sauerland and colleagues [16] also state that very few eyewitness studies (in contrast to neurological studies) have investigated stress with objective measures, relying instead on subjective assessments. This further complicates the comparison between 2D and real life, leaving a gap in the field that has not been sufficiently investigated. Future research should both include objective physiological measure of stress in threatening scenarios and investigate the potential of using 360-degree videos in VR to achieve more realistic events that can elicit reactions that may be more comparable to real life. Future research should also compare 2D, VR, and real-life using objective physiological measures to estimate possible differences between methods. The inclusion of a manipulation check of the sense of presence and perceived realism would also be of use when comparing 2D, VR, and real-life.

## Conclusion

The relevance of the current study is that it is novel and timely comparisons between alternative methods of investigating estimator variables on eyewitness accuracy. There is a clear need to experimentally investigate the differences between real life setting and laboratory-based studies, and although this study was not a direct comparison between live and 2D it was a comparison between a potentially much more advantageous approach compared to 2D. Our results did not lend support to the notion that VR results in higher identification or rejection accuracy, however this does not mean that the method is flawed but rather that more research is needed since the lower degree of accuracy might represent a true effect. We did find support for our hypotheses that accuracy is higher in threatening scenes (compared to non-threatening scenes) and that this is only the case in VR and not in 2D; suggesting that perhaps VR is better suited for an investigation of such manipulations. However, there is need for larger sample sizes and objective measures of stress (that we did not include in this study), but this study still represents an important first step into a new method of investigating eyewitness accuracy.

## Supporting information

S1 Appendix Eyewitness identification in virtual reality. (PDF)
