# Supplementary material for: Eyewitness identifications after witnessing threatening and non-threatening scenes in 360-degree virtual reality (or 2D) from first and third person perspectives
Source: PLoS One. 2020 Sep 2;15(9):e0238292. doi: 10.1371/journal.pone.0238292 (PMC7467270; doi:10.1371/journal.pone.0238292)
Supplement: S1 Appendix — (PDF) [file pone.0238292.s001.pdf]

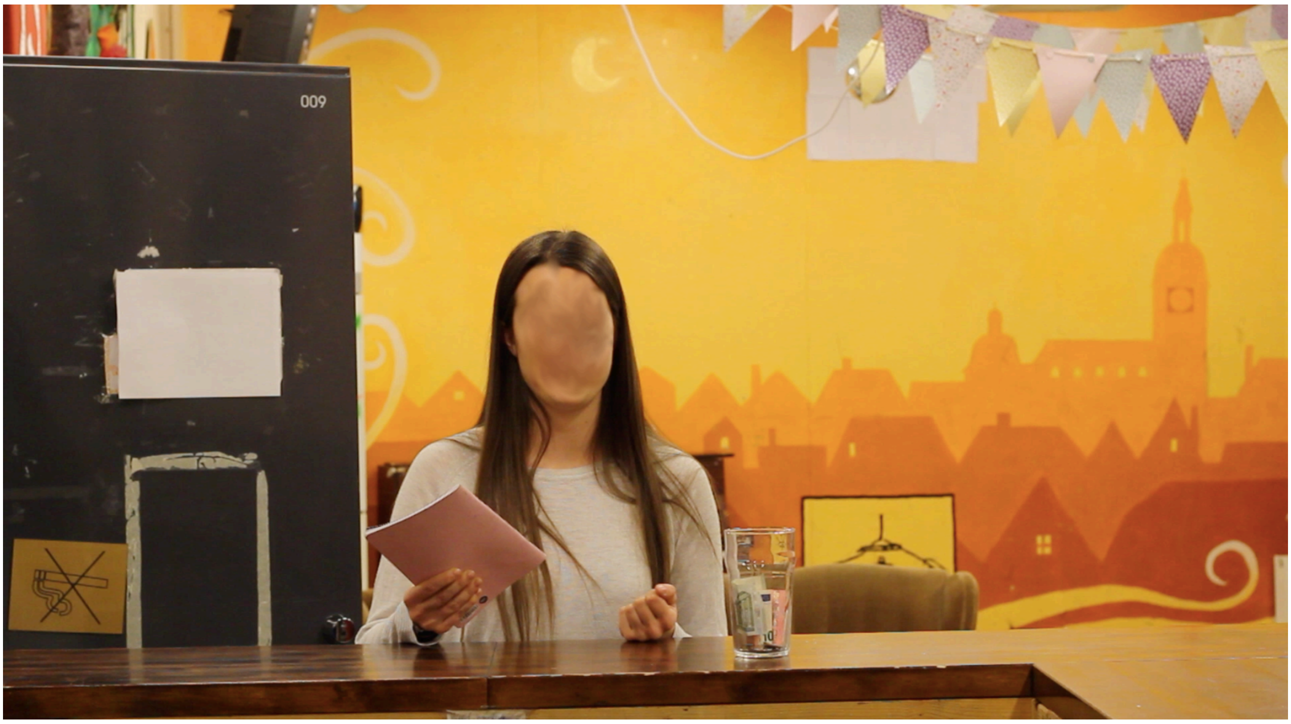

Figure 1. A still photo from a pre-recording (Canon camera) of one of the first-person non-threatening scenes used in our experiment. In this scene a female target is holding a booklet in their hand.

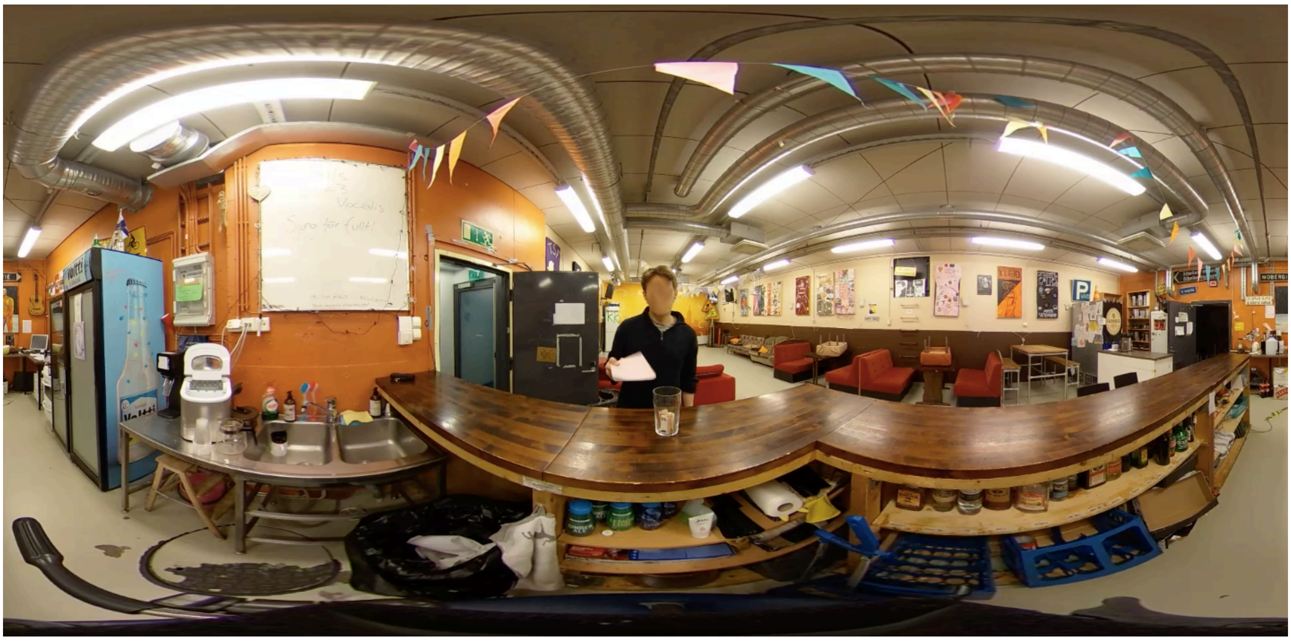

Figure 2. A still photo from a pre-recording (360-degree camera) of one of the first-person non-threatening scenes used in our experiment. In this scene a male target is holding a booklet in their hand. The video file has been converted so that the 360-degree viewpoint can be shown in 2D. The viewpoint is closer when viewing the event with a head mounted display in virtual reality.

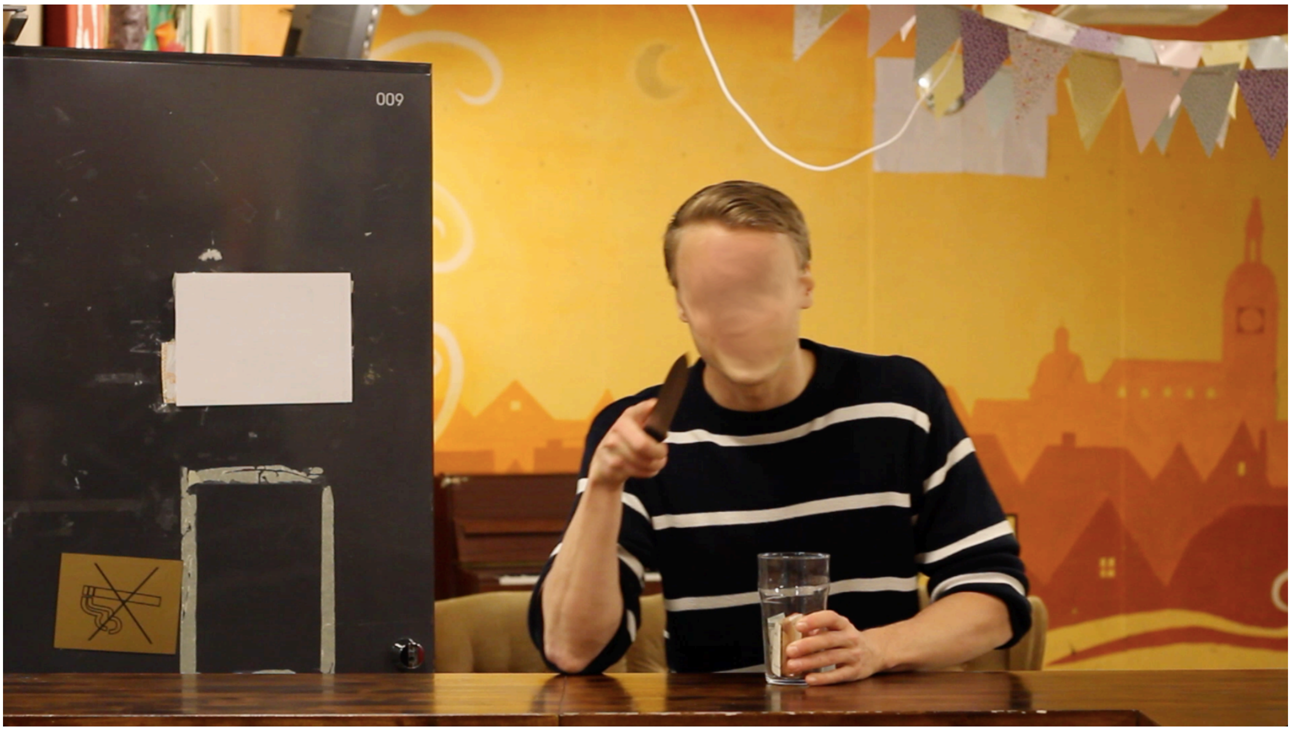

Figure 3. A still photo from a pre-recording (Canon camera) of one of the first-person threatening scenes used in our experiment. In this scene a male target is holding a booklet in their hand.

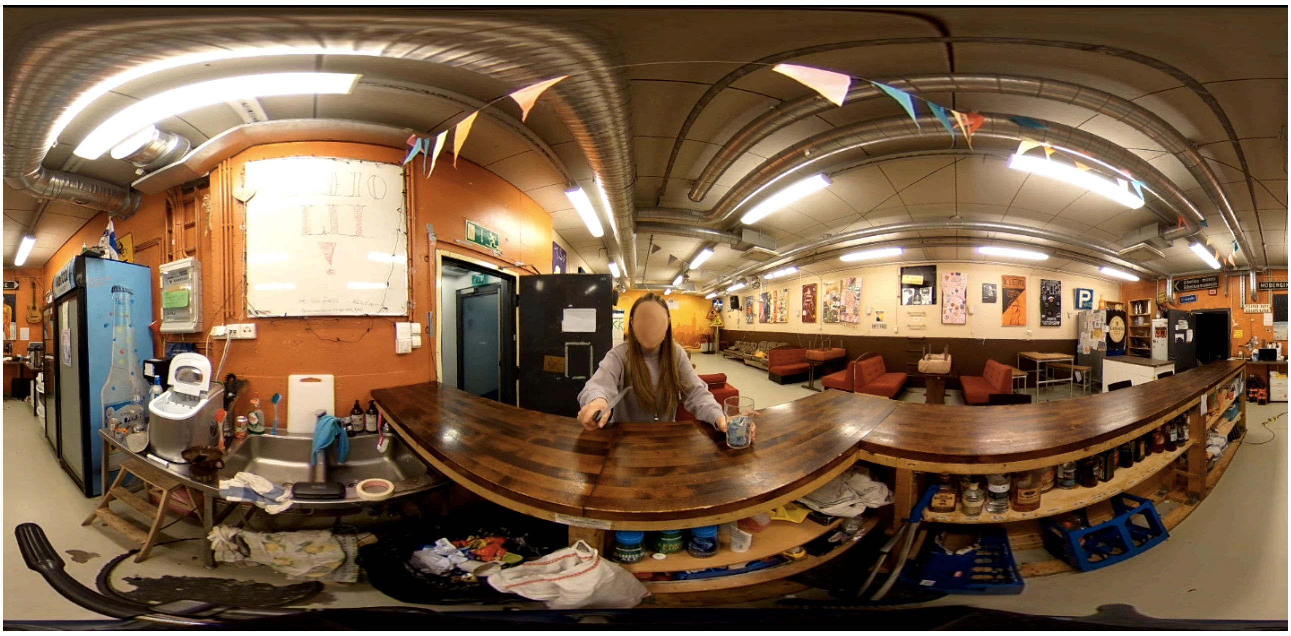

Figure 4. A still photo from a pre-recording (360-degree camera) of one of the first-person threatening scenes used in our experiment. In this scene a female target is holding a knife in their hand. The video file has been converted so that the 360-degree viewpoint can be shown in 2D. The viewpoint is closer when viewing the event with a head mounted display in virtual reality.

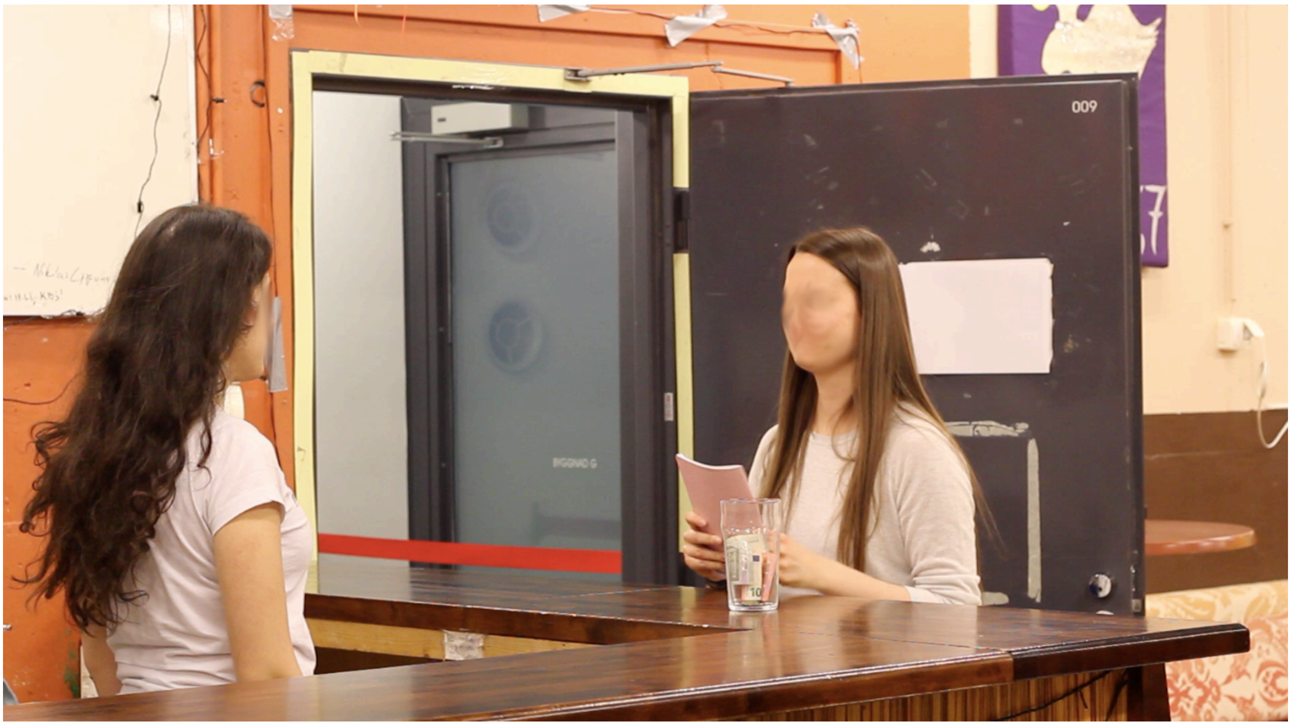

Figure 5. A still photo from a pre-recording (Canon camera) of one of the third-person non-threatening scenes used in our experiment. In this scene a female target is holding a booklet in their hand.

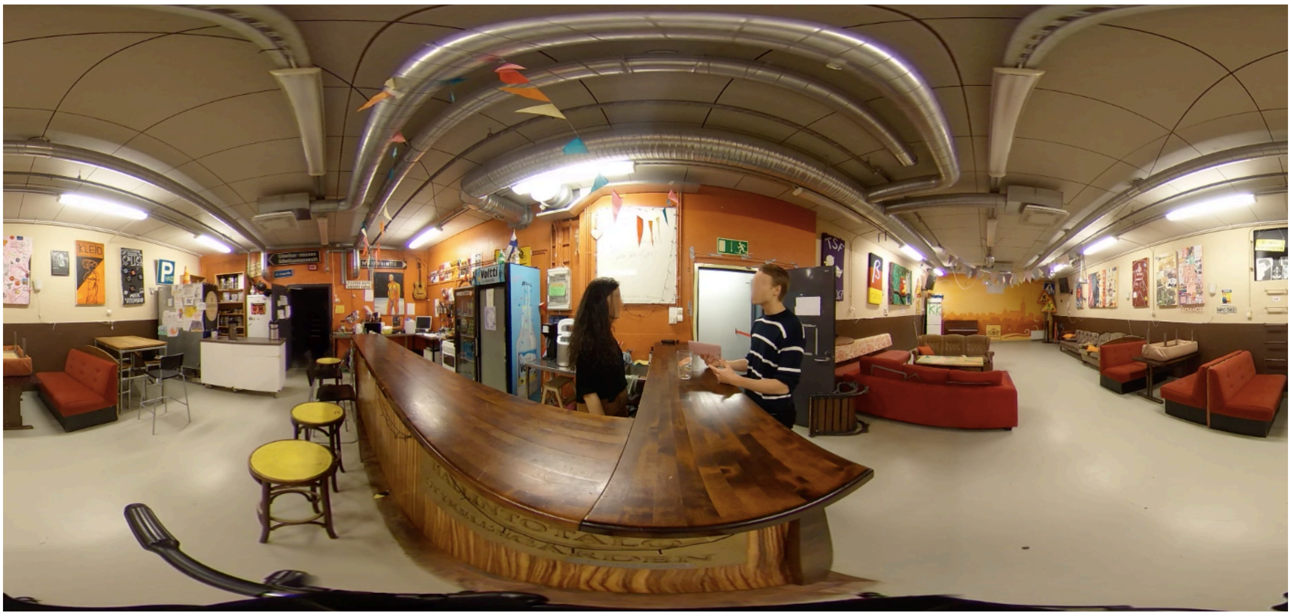

Figure 6. A still photo from a pre-recording (360-degree camera) of one of the third-person non-threatening scenes used in our experiment. In this scene a male target is holding a booklet in their hand. The video file has been converted so that the 360-degree viewpoint can be shown in 2D. The viewpoint is closer when viewing the event with a head mounted display in virtual reality.

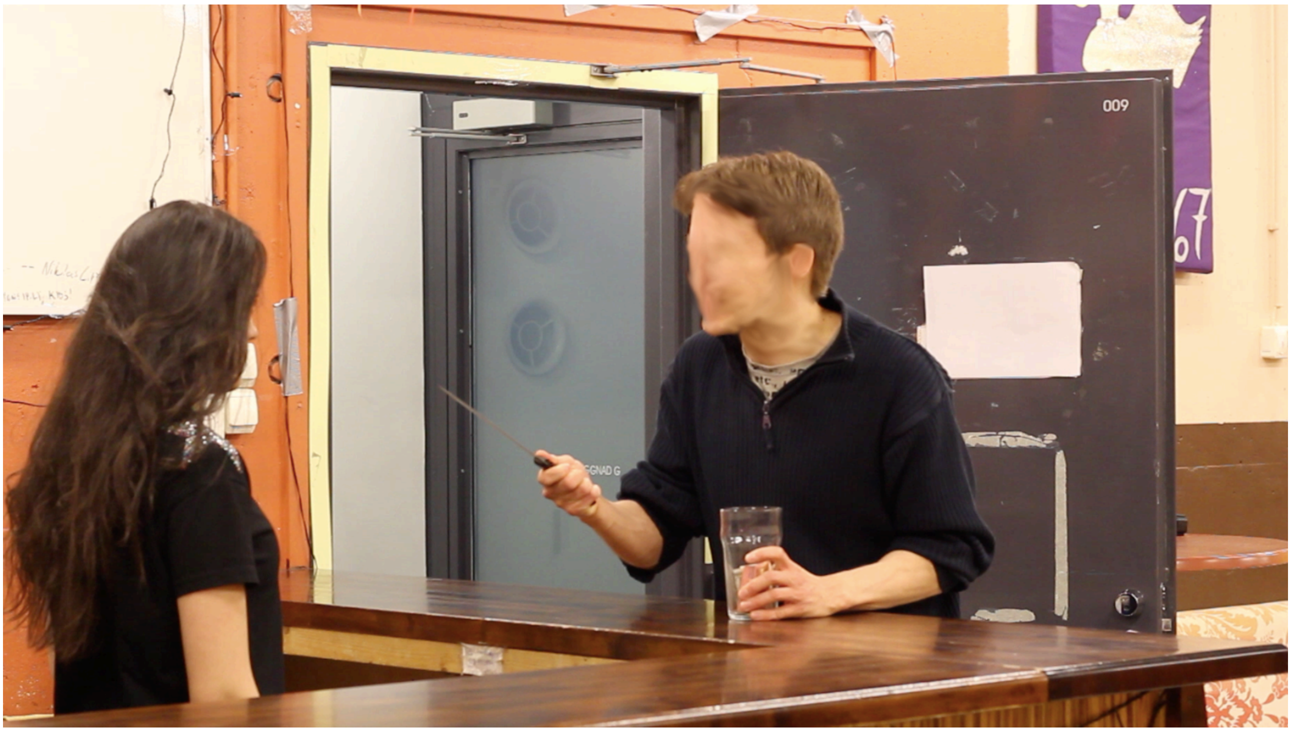

Figure 7. A still photo from a pre-recording (Canon camera) of one of the third-person threatening scenes used in our experiment. In this scene a male target is holding a knife in their hand.

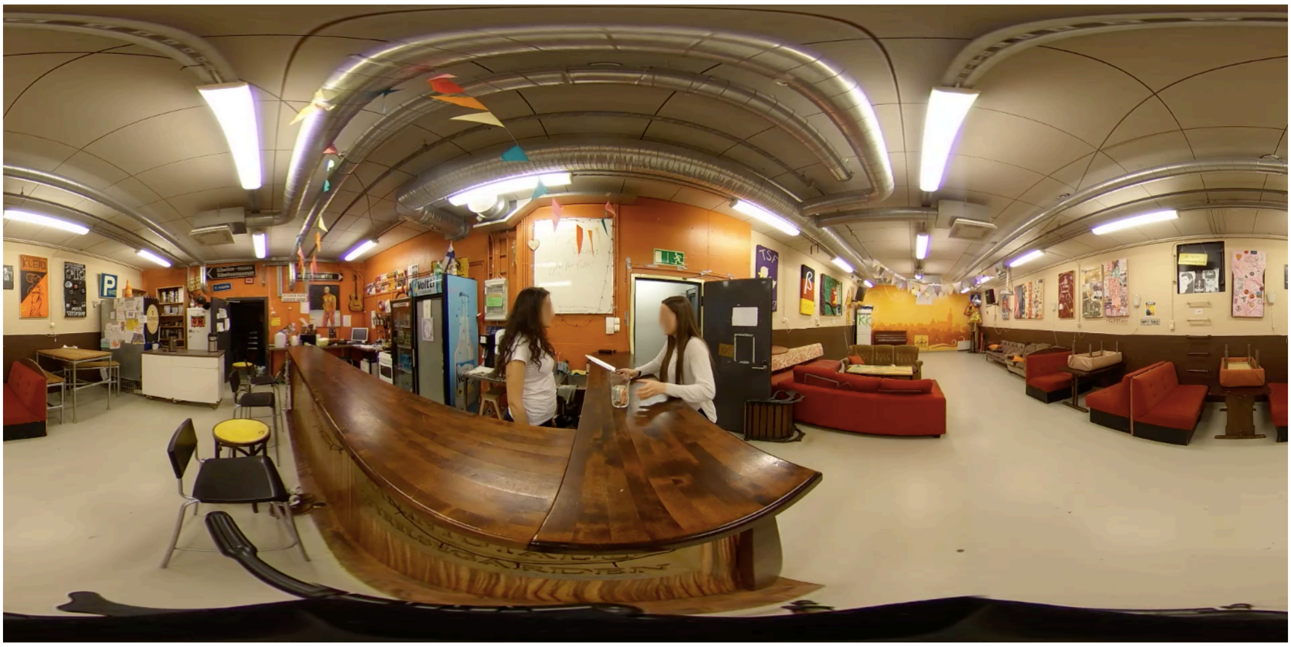

Figure 8. A still photo from a pre-recording (360-degree camera) of one of the third-person threatening scenes used in our experiment. In this scene a female target is holding a knife in their hand. The video file has been converted so that the 360-degree viewpoint can be shown in 2D. The viewpoint is closer when viewing the event with a head mounted display in virtual reality.
